# Transition patterns of metabolism-weight phenotypes over time: A longitudinal study using the multistate Markov model in China

**DOI:** 10.3389/fpubh.2022.1026751

**Published:** 2022-12-15

**Authors:** Hongya Zhang, Xiao Tang, Dongmei Hu, Guorong Li, Guirong Song

**Affiliations:** ^1^Department of Health Statistics, School of Public Health, Dalian Medical University, Dalian, Liaoning, China; ^2^Personnel Division, Gansu Provincial Hospital, Lanzhou, Gansu, China

**Keywords:** obesity, metabolic status, longitudinal study, multistate Markov model, China

## Abstract

**Background:**

A change in weight or metabolic status is a dynamic process, yet most studies have focused on metabolically healthy obesity (MHO) and the transition between MHO and metabolically unhealthy obesity (MUO); therefore, they have not fully revealed the nature of all possible transitions among metabolism-weight phenotypes over the years.

**Methods:**

This was a longitudinal study based on a retrospective health check-up cohort. A total of 9,742 apparently healthy individuals aged 20–60 years at study entry were included and underwent at least two health check-ups. Six metabolism-weight phenotypes were cross-defined by body mass index (BMI) categories and metabolic status as follows: metabolically healthy normal weight (MHNW), metabolically healthy overweight (MHOW), MHO, metabolically unhealthy normal weight (MUNW), metabolically unhealthy overweight (MUOW), and MUO. A multistate Markov model was used to analyse all possible transitions among these phenotypes and assess the effects of demographic and blood indicators on the transitions.

**Results:**

The transition intensity from MUNW to MHNW was the highest (0.64), followed by the transition from MHO to MUO (0.56). The greatest sojourn time appeared in the MHNW state (3.84 years), followed by the MUO state (2.34 years), and the shortest sojourn time appeared in the MHO state (1.16 years). Transition intensities for metabolic improvement gradually decreased with BMI level as follows: 0.64 for MUNW to MHNW, 0.44 for MUOW to MHNW, and 0.27 for MUO to MHO; however, transition intensities for metabolic deterioration, including MHNW to MUNW, MHOW to MUOW, and MHO to MUO, were 0.15, 0.38, and 0.56, respectively. In the middle-aged male group, elevated alanine aminotransferase (ALT), aspartate aminotransferase (AST), and uric acid (UA) increased the risk of deterioration in weight and metabolic status and decreased the possibility of improvement.

**Conclusion:**

Maintaining a normal and stable BMI is important for metabolic health. More attention should be given to males and elderly people to prevent their progression to an unhealthy metabolic and/or weight status. MHO is the most unstable phenotype and is prone to convert to the MUO state, and individuals with abnormal ALT, AST and UA are at an increased risk of transitioning to an unhealthy weight and/or metabolic status; therefore, we should be alert to abnormal indicators and MHO. Intervention measures should be taken early to maintain healthy weight and metabolic status.

## Introduction

Obesity defined by body mass index (BMI) has been a well-demonstrated causal factor for many diseases, such as metabolic syndrome (MetS), cardiovascular diseases (CVDs), type 2 diabetes mellitus (T2DM) and certain types of cancer (1–5), and consequently contributes to a reduced life expectancy and heavy social and economic burdens worldwide. Nevertheless, obese individuals exhibit heterogeneous phenotypes in their metabolic profiles, which usually involve cardio-metabolic risk factors. Thereby, metabolically healthy obesity (MHO) was put forward to define such a phenotype of obesity without elevated blood pressure, abnormal lipid profiles or low insulin sensitivity (6, 7). MHO is seemingly favourable to obesity-related chronic diseases compared to metabolically unhealthy obesity (MUO), but at present, MHO is usually considered a relatively unstable phenotype and is likely to deteriorate into MUO over time (8–10); notably, this deterioration can increase the risk of T2DM and CVDs (10–12).

In addition, there are also metabolically healthy overweight (MHOW) and metabolically unhealthy overweight (MUOW) phenotypes, which might increase the risk of CVDs compared with those with a metabolically healthy normal weight (MHNW) phenotype (13–15). Additionally, individuals with normal weight exhibit heterogeneous metabolic phenotypes, and a proportion of them have unhealthy metabolic profiles, described as metabolically unhealthy normal weight (MUNW) (15, 16). Individuals with MUNW often present with abnormal body fat distribution and obesity-related complications (e.g., hypertriglyceridaemia and insulin resistance) or a higher risk of T2DM and CVDs than those with MHNW (13, 14).

In fact, due to weight loss or weight gain, individuals may experience the transitions between different weight states over a period of time, involving normal weight, overweight and obesity (17). Likewise, the individual in a certain weight status may also experience improvement in metabolic profile, as well as metabolic deterioration or maintenance of one's initial status. Therefore, the change in weight and/or metabolic status is a dynamic process. However, to the best of our knowledge, the majority of previous studies only focused on the single transition between MHO and MUO (8–12). Because these studies ignored other possible transitions between these metabolism-weight phenotypes, defined simultaneously by weight and metabolic status, they did not fully reveal the nature of transitions between metabolism-weight phenotypes. To date, few studies have focused on all possible transitions between these phenotypes (14, 18). Only Kabat et al. explored the change in phenotypes as a stochastic process by Markov chain analysis, but this study was limited to postmenopausal females (18).

The multistate Markov model is a useful tool for describing a process in which an individual moves through a series of states in continuous time (19). This model can overcome the defects of traditional methods, such as logistic regression and Cox models, which can only consider a single outcome for longitudinal data. Overall, the model can fully take into account all possible states or outcomes, as well as the time and influencing factors of all possible transitions between states in a study, thereby describing the change process of disease in more detail, finding significant predictors on a certain transition, and estimating the intensity and probability of the transition in a specific population. Moreover, the model does not require the same time interval between two continuous observations for different individuals, allowing bidirectional reversible transition between states (i.e., deterioration or improvement) and imprecise time of the change between states.

Taken together, we used a multistate Markov model to explore the rules of possible transitions among metabolism-weight phenotypes over years in a health check-up cohort. Meanwhile, the effects of certain factors on the transitions were analysed. It is essential to clarify the nature of transitions among these phenotypes and determine which phenotypes are more prone to transit and the factors that influence the transition as a whole. Subsequently, it can be targeted to prevent individuals from progressing to a more severe phenotype, thereby reducing the risk of adverse outcomes to a certain extent.

## Materials and methods

### Study design and subjects

This was a retrospective longitudinal cohort study evaluating the data on health check-ups from January 2010 to December 2017 at the centre of the Second Hospital Affiliated with Dalian Medical University.

Data from each subject's initial health check-up were defined as the baseline data. Initially, 10751 subjects were included based on the following criteria: (1) BMI ≥ 18.5 kg/m^2^; (2) aged between 20 and 60 years at baseline; (3) no history of diabetes, CVDs, cerebrovascular diseases, viral hepatitis, liver cirrhosis, autoimmune liver disease, renal disease or rheumatic disease at baseline; (4) no missing data related to the study, including metabolic components, alanine aminotransferase (ALT), aspartate aminotransferase (AST), uric acid (UA) and serum creatinine (SCr) at baseline; and (5) underwent at least two health check-ups. Next, 636 subjects with a BMI < 18.5 kg/m^2^ and 373 subjects who were diagnosed with any of the above-mentioned diseases during follow-up were excluded. Finally, 9,742 subjects were included in our study. The methods of data collection in this study have been reported in detail previously (17, 20).

### Definitions

**Figure 1:**
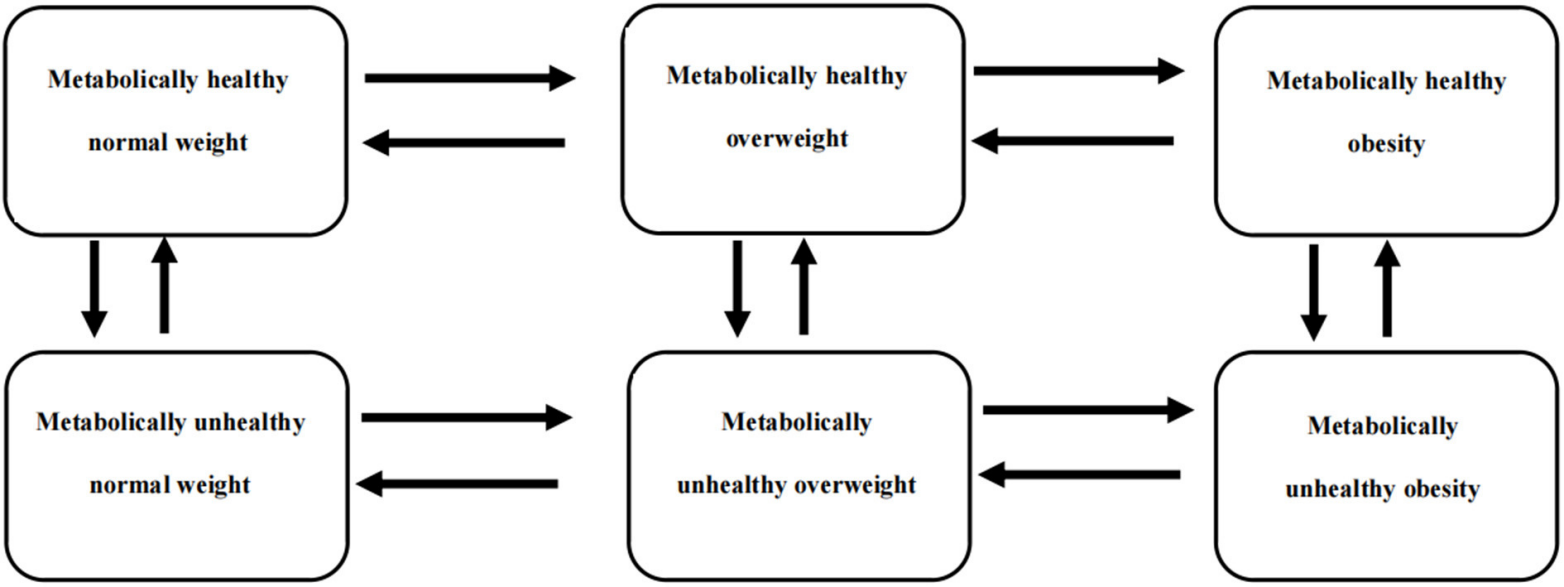
A six-state Markov model was used to describe the transition patterns among different metabolism-weight phenotypes, which were metabolically healthy normal weight (MHNW), metabolically healthy overweight (MHOW), metabolically healthy obesity (MHO), metabolically unhealthy normal weight (MUNW), metabolically unhealthy overweight (MUOW), and metabolically unhealthy obesity (MUO).

Weight status was classified according to BMI categories based on Chinese guidelines (21), and metabolic status, except abdominal obesity, was classified according to the revised National Cholesterol Education Project Adult Treatment Panel (NCEP ATP III) criteria (22). Cross-classification of weight and metabolic status in Table 1 created six metabolism-weight phenotypes (Figure 1).

**Table 1:** Definitions of the categories of weight, metabolism, age, ALT, AST, SCr and UA.

**Categories**		**Definitions**
Weight	Normal weight	18.5kg/m^2^ ≤ BMI < 24 kg/m^2^
	Overweight	24 kg/m^2^ ≤ BMI < 28 kg/m^2^
	Obesity	BMI ≥ 28 kg/m^2^
Metabolism	Metabolically unhealthy	Subjects who have two or more of the following four criteria are considered metabolically unhealthy. 1. Elevated blood pressure: SBP ≥ 130 mHg/DBP ≥ 85 mmHg and/or patients who have been diagnosed as hypertension and received anti-hypertensive medication; 2. Elevated blood glucose: FPG ≥ 5.6 mmol/L (100 mg/dL) and/or 2hPG≥7.8 mmol/L (140 mg/dL) and/or patients who have been diagnosed with diabetes and received hypoglycemic medication; 3. Increased triglyceride, TG ≥ 1.7 mmol/L (150 mg/dL); 4. Low high-density lipoprotein cholesterol, male HDL-C < 1.0 mmol/L (40 mg/dL), female HDL-C < 1.3 mmol/L (50 mg/dL).
	Metabolically healthy	Subjects who have only one or none of the upper four criteria are considered metabolically healthy.
Age	Young group	≤ 45 years old
	Middle-aged group	> 45 years old
ALT	Elevated group	ALT > 40U/L
	Normal group	ALT ≤ 40U/L
AST	Elevated group	AST > 40U/L
	Normal group	ALT ≤ 40U/L
SCr	Elevated group	Male SCr > 133 μmol/L, female SCr > 106 μmol/L
	Normal group	Male SCr ≤ 133 μmol/L, female SCr ≤ 106 μmol/L
UA	Elevated group	Male UA > 420μmol/L, female UA > 360μmol/L
	Normal group	Male UA ≤ 420μmol/L, female UA ≤ 360μmol/L

BMI, body mass index; SBP, systolic blood pressure; DBP, diastolic blood pressure; FPG, fasting plasma glucose; 2hPG, 2-hour postprandial blood glucose; TG, triglycerides; HDL-C, high-density lipoprotein cholesterol; ALT, alanine aminotransferase; AST, aspartate aminotransferase; SCr, serum creatinine; UA, uric acid.

Considering that perimenopause and menopause might cause some metabolic change for females and that the average age of females entering perimenopause is approximately 45 years old (23), two age groups were defined as follows: the young group (≤45 years old) and the middle-aged group (>45 years old). Elevated ALT, AST, SCr, and UA were defined according to their usual reference values for Chinese adults, as shown in Table 1.

### Statistical analysis

The basic analysis was performed by IBM SPSS Statistics 25.0 (IBM Corp). Medians and quartiles were used to describe variables with non-normal distributions. Percentages were used to describe categorical variables.

The MSM package R 4.0.2 was used to build the continuous multistate Markov model. As shown in Figure 1, 6 states were mutually exclusive, and there was no absorbing state because the mortality rate among the healthy subjects was very low. Notably, instantaneous transitions were only permitted from adjacent states in the continuous-time Markov model, so it was postulated that an individual in a certain state could only transition to his or her adjacent states horizontally or vertically in a very short time, as shown in Figure 1. The transition intensities (*q*_*rs*_) between states and the mean sojourn time in each state were estimated, and *q*_*rs*_(r ≠ s) can be used to represent the instantaneous risk of moving from state *r* to state *s* at time *t*.


$$\begin{array}{l} q_{rs}=\underset{\delta t\to 0}{\lim}\frac{p\left(s\left(t+\delta t\right)=s|S\left(t\right)=r\right)}{\delta t} \end{array}$$

In addition, the effects of covariates on a particular transition intensity were estimated by modelling the intensity as a function of these variables similar to a proportional hazards model.


$$\begin{array}{l} q_{rs}\left(z\left(t\right)\right)=q_{rs}\left(0\right)\exp\left(\beta_{rs}^{T}z\left(t\right)\right) \end{array}$$

In our study, the effects of covariates on transitions were assessed, including sex, age group, ALT, AST, SCr and UA. A detailed explanation of the model has been reported in previous studies (17, 19). Excel 365 was used to make line graphs with predicted probabilities of transitions from each phenotype to any other phenotype over 6 years.

Sensitivity analyses were completed to describe the patterns of the transitions among six phenotypes in four subgroups based on sex and age group to demonstrate the robustness of the results.

## Results

### The characteristics of each phenotype at baseline

A total of 9,742 individuals aged 20–60 years at baseline, including 4,650 (47.73%) males and 5,092 (52.27%) females, constituted the final analytic dataset. The average follow-up time was 3.66 (1.87–5.03) years, and the length of time between two adjacent check-ups was approximately 1 year. Table 2 presents the baseline characteristics among the six phenotypes. At baseline, there were 4,387 individuals (45.03%) in MHNW, 1,761 (18.08%) in MHOW, 351 (3.60%) in MHO, 918 (9.42%) in MUNW, 1,552 (15.93%) in MUOW and 773 (7.93%) in MUO. Moreover, 33.29% of the total study population was metabolically unhealthy. A total of 68.77% of the obese individuals were metabolically unhealthy, and 46.85% of the overweight individuals were metabolically unhealthy, whereas only 15.10% of the normal-weight individuals were metabolically unhealthy.

**Table 2:** The characteristics of each metabolism-weight phenotype at baseline.

**Baseline characteristics**	**MHNW**	**MHOW**	**MHO**	**MUNW**	**MUOW**	**MUO**
	**(*n =* 4,387, 45.03%)**	**(*n =* 1,761, 18.08%)**	**(*n =* 351, 3.60%)**	**(*n =* 918, 9.42%)**	**(*n =* 1,552, 15.93%)**	**(*n =* 773, 7.93%)**
Age(years)	34.00 (28.00, 42.00)	39.00 (30.00, 46.50)	36.00 (30.00, 44.00)	41.00 (32.00, 48.00)	43.00 (36.00, 50.00)	41.00 (32.00, 48.00)
Male (%)	3,216 (73.31)	741 (42.08)	102 (29.06)	486 (52.94)	403 (25.97)	144 (18.63)
BMI(kg/m^2^)	21.48 (20.31, 22.66)	25.43 (24.62, 26.40)	29.07 (28.41, 30.41)	22.55 (21.45, 23.34)	25.80 (24.92, 26.79)	29.41 (28.67, 30.86)
SBP(mmHg)	114.00 (107.00, 122.00)	120.00 (113.00, 127.00)	124.00 (118.00, 131.00)	130.00 (117.00, 136.00)	132.00 (123.00, 138.00)	134.00 (127.00, 140.00)
DBP(mmHg)	69.00 (64.00, 76.00)	73.00 (67.00, 79.00)	77.00 (70.00, 82.00)	78.00 (70.00, 85.25)	81.00 (74.00, 88.00)	84.00 (77.00, 89.50)
FPG(mmol/L)	5.18 (4.95, 5.43)	5.33 (5.04, 5.54)	5.33 (5.11, 5.55)	5.73 (5.43, 6.00)	5.77 (5.48, 6.12)	5.74 (5.39, 6.14)
TC(mmol/L)	4.53 (4.05, 5.07)	4.76 (4.20, 5.28)	4.80 (4.29, 5.31)	4.72 (4.19, 5.43)	4.92 (4.42, 5.53)	4.93 (4.41, 5.57)
TG(mmol/L)	0.80 (0.62, 1.04)	1.02 (0.78, 1.33)	1.21 (0.92, 1.50)	1.32 (0.89, 1.95)	1.76 (1.21, 2.29)	1.90 (1.42, 2.46)
HDL-C(mmol/L)	1.41 (1.24, 1.61)	1.25 (1.09, 1.44)	1.13 (1.03, 1.30)	1.16 (0.99, 1.29)	1.07 (0.93, 1.23)	1.01 (0.90, 1.15)
LDL-C(mmol/L)	2.52 (2.11, 2.98)	2.82 (2.37, 3.32)	2.98 (2.49, 3.44)	2.79 (2.33, 3.29)	2.95 (2.50, 3.41)	2.94 (2.58, 3.45)
ALT(U/L)	15.00 (12.00, 21.00)	21.00 (16.00, 30.00)	28.00 (20.00, 43.00)	19.50 (15.00, 27.00)	27.00 (19.00, 39.00)	36.00 (25.00, 55.00)
AST(U/L)	19.00 (17.00, 23.00)	21.00 (18.00, 25.00)	23.00 (19.00, 27.00)	21.00 (18.00, 25.00)	22.00 (19.00, 27.00)	25.00 (20.00, 33.00)
SCr(mg/dL)	59.00 (52.00, 69.00)	70.00 (57.05, 81.00)	72.00 (60.00, 80.00)	65.00 (55.00, 75.00)	73.00 (63.00, 81.00)	74.00 (65.00, 82.00)
UA(μmol/L)	266.15 (227.80, 320.00)	329.00 (271.00, 393.00)	371.00 (321.00, 421.00)	312.00 (257.50, 372.00)	361.00 (308.57, 416.00)	393.00 (342.00, 454.56)

Data are presented as median (P_25_, P_75_) for continuous variables and percentage for categorical variables. MHNW, metabolically healthy normal weight; MHOW, metabolically healthy overweight; MHO, metabolically healthy obesity; MUNW, metabolically unhealthy normal weight; MUOW, metabolically unhealthy overweight; MUO, metabolically unhealthy obesity. BMI, body mass index; SBP, systolic blood pressure; DBP, diastolic blood pressure; FPG, fasting plasma glucose; TC, total cholesterol; TG, triglycerides; HDL-C, high-density lipoprotein cholesterol; LDL-C, low-density lipoprotein cholesterol; ALT, alanine aminotransferase; AST, aspartate aminotransferase; SCr, serum creatinine; UA, uric acid.

### The observed transition frequency, estimated transition intensity and mean sojourn time

The frequency of transitions observed during the study is displayed in Table 3. In total, most individuals maintained their original phenotypes during follow-up. For instance, 80.20% of those in MHNW and 68.46% of those in MUO maintained their original phenotype, while only 46.10% of those in MHO stayed in their preceding phenotypes. Additionally, 30.80% of those in MHO progressed to MUO, 20.41% of those in MHOW progressed to MUOW, and only 9.29% of those in MHNW progressed to MUNW. In contrast, 37.80% of those in MUNW regressed to MHNW, 23.21% of those in MUOW regressed to MHOW, and only 14.20% of those in MUO regressed to MHO.

**Table 3:** The observed transition frequency during about 1 year in the study [*n* (%)].

**Original phenotype**	**Follow-up phenotype**					
	**MHNW**	**MHOW**	**MHO**	**MUNW**	**MUOW**	**MUO**
**MHNW**	9,234 (80.20)	877(7.62)	23(0.20)	1,069 (9.29)	278 (2.41)	32 (0.28)
**MHOW**	660 (13.25)	2,904 (58.29)	192 (3.85)	107 (2.15)	1,017 (20.41)	102 (2.05)
**MHO**	18 (1.85)	149 (15.30)	449 (46.10)	4 (0.41)	54 (5.54)	300 (30.80)
**MUNW**	943 (37.80)	148 (5.93)	3 (0.12)	1,164 (46.65)	231 (9.26)	6 (0.24)
**MUOW**	251 (6.15)	948 (23.21)	68 (1.67)	201 (4.92)	2,380 (58.28)	236 (5.78)
**MUO**	28 (1.45)	104 (5.37)	275 (14.20)	7 (0.36)	197 (10.17)	1,326 (68.46)

MHNW, metabolically healthy normal weight; MHOW, metabolically healthy overweight; MHO, metabolically healthy obesity; MUNW, metabolically unhealthy normal weight; MUOW, metabolically unhealthy overweight; MUO, metabolically unhealthy obesity.

**Figure 2:**
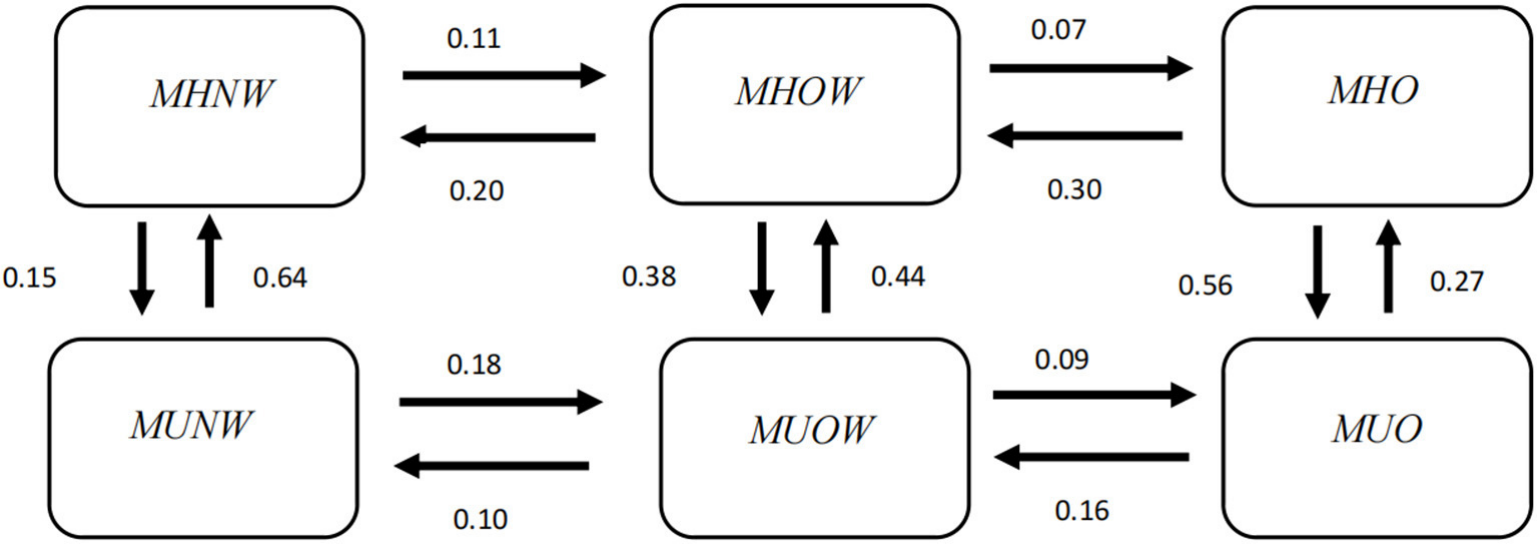
Transition intensity from one phenotype to another estimated by the multistate Markov model. MHNW, metabolically healthy normal weight; MHOW, metabolically healthy overweight; MHO, metabolically healthy obesity; MUNW, metabolically unhealthy normal weight; MUOW, metabolically unhealthy overweight; MUO, metabolically unhealthy obesity.

The transition intensities estimated by the multistate Markov model are presented in Supplementary Table 1. In all possible transitions, the transition intensity from MUNW to MHNW was the highest (0.64), followed by MHO to MUO (0.56). In Figure 2, the downwards transition intensity, namely, metabolic deterioration, gradually increased from left to right, from 0.15 (MHNW → MUNW) to 0.56 (MHO → MUO), whereas the upwards transition intensity, namely, metabolic improvement, gradually decreased from left to right, from 0.64 (MUNW → MHNW) to 0.27 (MUO → MHO). These results illustrated that individuals with lower BMI levels were more likely to experience metabolic improvement, whereas individuals with higher BMI levels were more likely to experience metabolic deterioration. Regarding horizontal transitions, most of the transitions towards decreasing BMI showed higher transition intensity than those towards increasing BMI, except the transition between MUNW and MUOW.

The greatest sojourn time appeared in the MHNW state (3.84 years), followed by the MUO state (2.34 years), and the shortest sojourn time appeared in the MHO state (1.16 years; Table 4), which indicated that the MHNW state was relatively stable, while MHO was the most unstable state and most prone to change.

**Table 4:** Estimated mean sojourn time in each phenotype.

**Phenotype**	**Estimated mean sojourn time (years)**	**Standard errors**	**95% CI**	
			**lower**	**upper**
**MHNW**	3.84	0.09	3.69	4.02
**MHOW**	1.56	0.04	1.48	1.63
**MHO**	1.16	0.06	1.06	1.28
**MUNW**	1.23	0.04	1.16	1.30
**MUOW**	1.59	0.04	1.51	1.68
**MUO**	2.34	0.11	2.15	2.56

MHNW, metabolically healthy normal weight; MHOW, metabolically healthy overweight; MHO, metabolically healthy obesity; MUNW, metabolically unhealthy normal weight; MUOW, metabolically unhealthy overweight; MUO, metabolically unhealthy obesity.

### Transition probabilities over 6 years predicted for each phenotype

**Figure 3:**
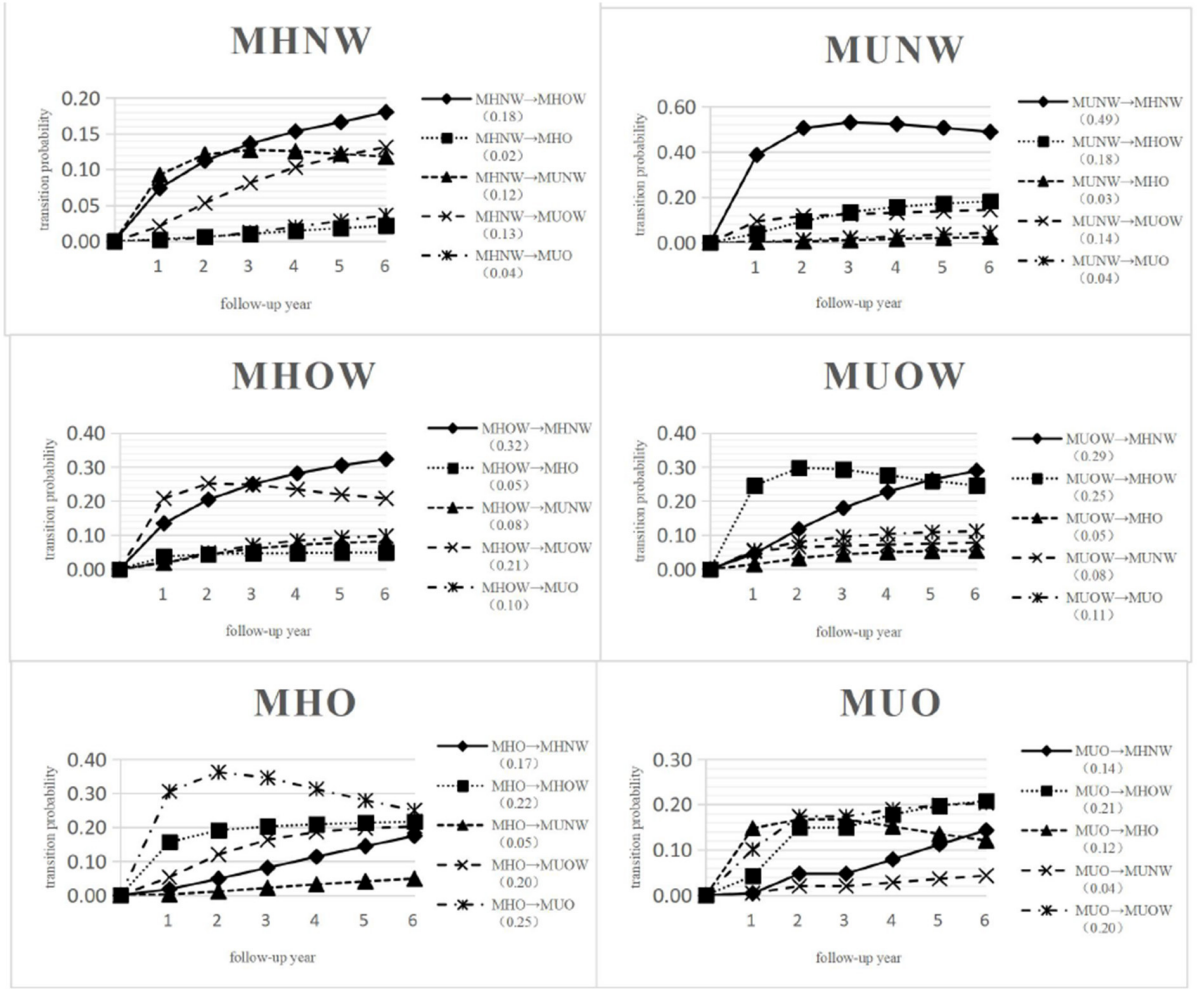
Predicted probabilities of transition from MHNW, MHOW, MHO, MUNW, MUOW, MUO to any other state over 6 years. MHNW, metabolically healthy normal weight; MHOW, metabolically healthy overweight; MHO, metabolically healthy obesity; MUNW, metabolically unhealthy normal weight; MUOW, metabolically unhealthy overweight; MUO, metabolically unhealthy obesity.

The predicted transition probabilities over 6 years from each state to the other states are given in Figure 3. Unlike the transition intensity shown in Figure 2 and Supplementary Table 1, which only represents the transition possibility between adjacent states within a very short period of time, the transition probability is the possibility of changing from a certain state to any other state over a period of time.

In Figure 3, 51.80% of those in MHNW, 24.00% of those in MHOW, 11.05% of those in MHO, 11.72% of those in MUNW, 22.20% of those in MUOW and 28.20% of those in MUO did not transition and remained in their initial states over 6 years. The highest transition probability was observed for MUNW to MHNW (0.49), followed by MHOW to MHNW (0.32); however, the lowest transition probability was observed for MHNW to MHO (0.02) after 6 years.

The probability for an individual in MHNW at baseline to transition to a metabolically abnormal state after 6 years was only 0.29. Specifically, the probability of transitioning from MHNW to MUOW was 0.13, MHNW to MUNW was 0.12, and MHNW to MUO was 0.04. An individual in MHOW at baseline was more likely to maintain a metabolically healthy state with a probability of 0.61, whereas the probability of transition to any metabolically abnormal state over 6 years was only 0.39. An individual in MHO at baseline had a fifty-fifty chance of maintaining a metabolically healthy state or progressing to a metabolically abnormal state after 6 years. An individual in MUNW at baseline was more likely to regress to a metabolically healthy state with a probability of 0.70 after 6 years. An individual in MUOW at baseline was more likely to regress to a metabolically healthy state with a probability of 0.59 after 6 years. An individual in MUO at baseline showed a probability 0.47 of regressing to a metabolically healthy state after 6 years.

### The effects of covariates on transitions

**Figure 4:**
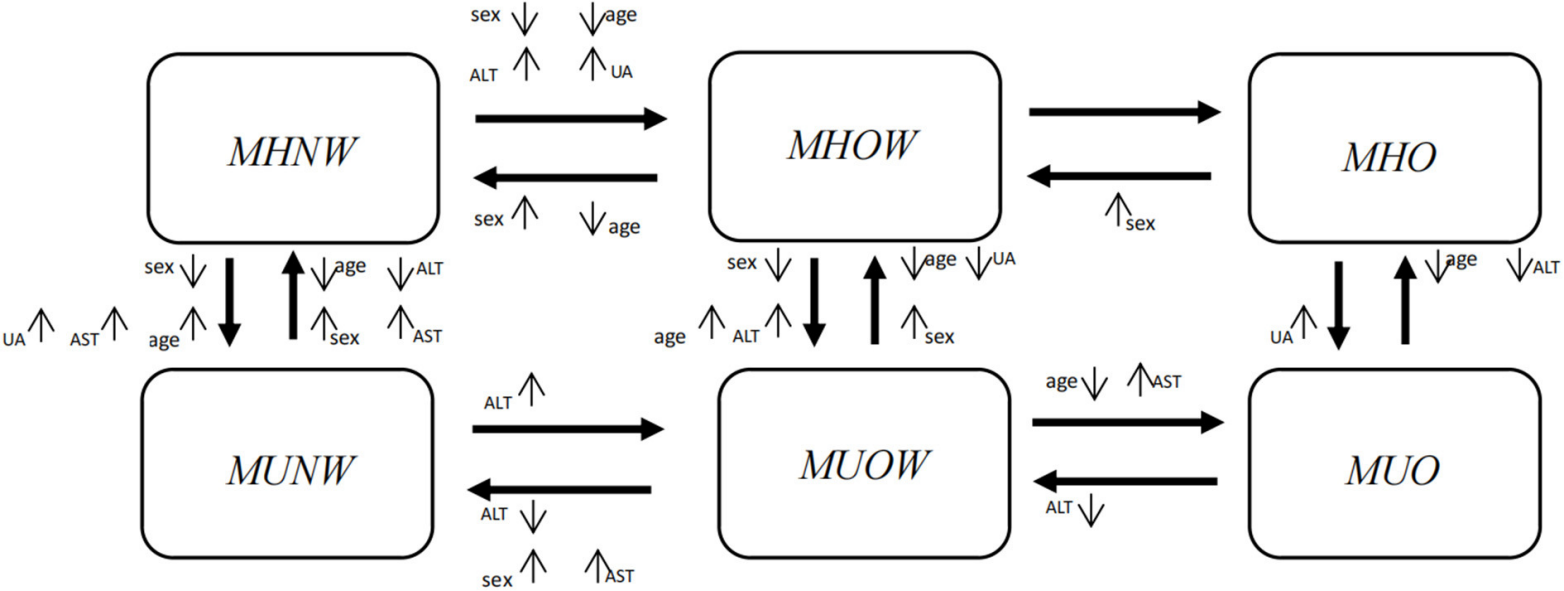
Factors showing the significant effects on transitions from one phenotype to another in a multiple variable analysis. Significant factors included sex, age, ALT, AST and UA. Sex, females vs. males; Age, middle-aged group (>45 years old) vs. young group (≤ 45 years old); ALT, elevated vs. normal; AST, elevated vs. normal; UA, elevated vs. normal. The direction of the arrow beside a factor denotes the significant impact of this factor on a certain transition. ↑means the factor increases the probability of the transition compared with the reference level; ↓means the factor decreases the probability of the transition compared with the reference level. ALT, alanine aminotransferase; AST, aspartate aminotransferase; UA, uric acid.

Figure 4 succinctly displays the effects of factors that were significantly associated with the transition from one adjacent phenotype to another, and Supplementary Table 2 displays these results in detail.

In Figure 4, horizontally, when individuals maintained their metabolic status, females were more likely than males to experience an improvement in their weight status (MHOW → MHNW, HR = 2.27, 95% CI: 1.90~2.71; MHO → MHOW, HR = 1.84, 95% CI: 1.32~2.56; MUOW → MUNW, HR = 1.84, 95% CI: 1.40~2.43), whereas females were less likely to progress with their weight status (MHNW → MHOW, HR = 0.63, 95% CI: 0.55~0.73). From the vertical perspective, when individuals maintained their weight status, females were more likely than males to experience an improvement in their metabolic status (MUNW → MHNW, HR = 1.19, 95% CI: 1.02~1.40; MUOW → MHOW, HR = 1.17, 95% CI: 1.00~1.37), whereas females were less likely to deteriorate in their metabolic status (MHNW → MUNW, HR = 0.68, 95% CI: 0.58~0.79; MHOW → MUOW, HR = 0.77, 95% CI: 0.66~0.90).

Horizontally, when individuals maintained their metabolic status, middle-aged individuals were less likely than young individuals to experience an improvement in their weight status, and they were less likely to progress with their weight status. From the vertical perspective, when individuals maintained their weight status, middle-aged individuals were more likely than young individuals to deteriorate to an abnormal metabolic status, whereas they were less likely to improve their metabolic status.

In general, when individuals maintained their metabolic status, those with elevated ALT, AST or UA levels were more likely to progress horizontally to a worse weight status than those with normal levels, and they were also less likely to improve their weight status. From the vertical perspective, when individuals maintained their weight status, those with elevated ALT, AST or UA levels were more likely to deteriorate to an abnormal metabolic status than those with normal levels, and they were also less likely to improve their metabolic status (Supplementary Table 2).

### Model assessment and sensitivity analysis

**Figure 5:**
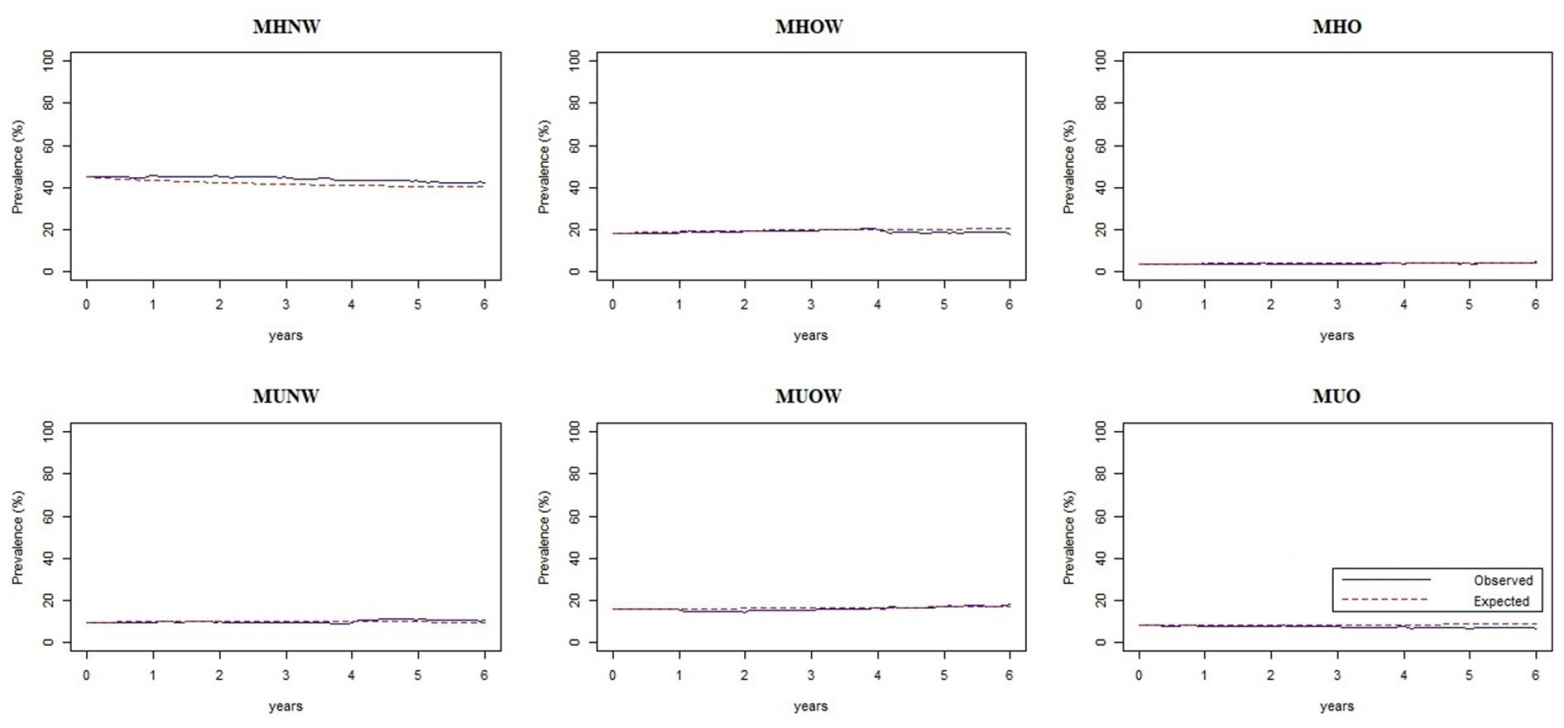
Assessment plots of the multistate Markov model showing the observed and expected percentages of each phenotype against time. MHNW, metabolically healthy normal weight; MHOW, metabolically healthy overweight; MHO, metabolically healthy obesity; MUNW, metabolically unhealthy normal weight; MUOW, metabolically unhealthy overweight; MUO, metabolically unhealthy obesity.

The observed and expected percentages for each phenotype were plotted against time (Figure 5). Figure 5 shows that the two curves of observed and expected percentages tended to coincide, indicating a good goodness-of-fit of the model.

The estimated transition intensities for each subgroup are presented in Supplementary Table 3, and the mean sojourn time for different phenotypes are presented in Supplementary Table 4. Overall, the four subgroups all presented the same transition patterns among the six phenotypes as the whole population. For instance, individuals in any subgroup with lower BMI levels were more likely to experience metabolic improvement, whereas individuals with higher BMI levels were more likely to experience metabolic deterioration. Regarding horizontal transitions, individuals with a certain metabolic status, whether normal or abnormal, were more likely to experience an improvement in body weight status; that is, they were more inclined to develop in the direction of BMI reduction. In the four subgroups, the greatest sojourn time nearly all appeared in the MHNW state, followed by the MUO state, and the shortest appeared in the MHO state.

## Discussion

In this longitudinal study of a cohort undergoing health check-ups annually, the patterns of the transitions among six metabolism-weight phenotypes were comprehensively described. According to the observed transition frequencies, the estimated transition intensities and predicted transition probabilities over 6 years, obese and overweight individuals in metabolic healthy status were more likely to experience metabolic deterioration than normal-weight individuals. On the other hand, it was more difficult to regress to a metabolically healthy status from a metabolically unhealthy status for obese and overweight individuals compared with normal-weight individuals. This finding was consistent with previous studies. Hamer et al. (9) found that 16.6% of those in MHNW progressed to MUNW, 26.2% of those in MHOW progressed to MUOW, and 44.5% of those in MHO progressed to MUO over 8 years of follow-up based on the English Longitudinal Study of Ageing. Kabat et al. (18) also revealed these features of the transitions in a cohort of postmenopausal females. Weight and metabolic status interact, and there is heterogeneity in the risk of abnormal metabolic status among individuals with different weight statuses (24). In the progression from normal weight to overweight and then to obesity, SBP, DBP, waist circumference and insulin resistance increased gradually, while HDL-C showed a downwards trend, which indicated a trend of metabolic deterioration (15). Thus, corresponding measures, such as dietary interventions and physical exercise, should be taken in time to maintain normal and stable weight status to prevent the occurrence of various metabolic disorders and further prevent related chronic diseases (25, 26).

The prevalence of the MHO phenotype (3.60%) at baseline in our study was lower than that of other phenotypes, and 30.80% of those in MHO progressed to MUO in ~1 year. The transition intensity from MHO to MUO (0.56) was the second highest among 14 possible transitions, and its mean sojourn time (1.16 years) was shortest. Moreover, an individual with MHO at baseline had a fifty-fifty chance of maintaining a metabolically healthy state or progressing to a metabolically abnormal state after 6 years. All these results intensely demonstrated that MHO is an unstable and transient state and that there is a strong possibility that individuals with MHO will become metabolically unhealthy. These findings were in line with other similar studies (8, 9, 11, 27–29). Recently, Song et al. (11) reported that 39.53% of the participants with initial MHO converted to MUO during a median follow-up of 10.1 years in China, and the conversion from metabolically healthy to unhealthy for obese people might increase the chance of developing diabetes compared to those with a stable metabolic healthy state. Bell et al. (28) and Eshtiaghi et al. (29) both have expressed the natural course of MHO progression to metabolic deterioration, and that those with MHO are at risk of future metabolic derangement. However, these studies described the change in MHO over time only based on observed frequency, and our study quantified the possibility of adjacent transitions based on a multistate Markov model; thus, our results comprehensively show the rules of possible transitions among six phenotypes.

Furthermore, our results show that MHNW and MUO are two relatively stable states, in contrast to MHO. In other words, MUO is an unfavourable state, and individuals with MUO are less likely to improve in metabolism and weight than MHO. Additionally, MHO is prone to progressing to MUO. Therefore, the prevention strategies implemented are also warranted for individuals with MHO, and measures, such as weight loss and therapeutic interventions, should be taken in a timely manner to prevent the development of MUO.

Unlike other studies, our study tested the effects of some factors on transitions in multiple variable analysis. First, males were generally more likely to deteriorate in weight or metabolic status than females, and females were more likely to improve in weight or metabolic status than males. Many studies have found that the incidence of MetS in males is higher than that in females; accordingly, males are thought to be more likely than females to suffer from multiple metabolic abnormalities. Tang et al. (17) found that males were more likely to become overweight and more resistant to recover from worse states than females. Moussa et al. (30) found that males were more likely than females to switch from metabolically normal to metabolically abnormal states, and sex was a significant predictor of metabolic health based on a study in the UK. This might be due to differences in genetics, oestrogen, lifestyle behaviours, or perceptions of weight between males and females.

Second, middle-aged individuals were less likely than young individuals to experience transitions in their weight status, regardless of progression or regression; that is, middle-aged individuals who were overweight or obese tended to maintain their preceding weight state and were less likely to regress to a normal weight. Therefore, maintaining normal weight early for young people could be easier and more effective than maintaining normal weight for middle-aged people. This finding was consistent with our previous study (17). Moreover, the middle-aged individuals were generally more likely than the young individuals to deteriorate to an abnormal metabolic status, whereas middle-aged individuals were less likely to improve their metabolic status. Moussa et al. (30) reported that an age of 50–60 years at baseline increased the relative risk of transitioning into a metabolically unhealthy status. Some research observed a decrease in the prevalence of MHO with increasing age, independent of sex (30, 31). Thus, the likelihood of maintaining metabolic health decreases with age. Collectively, perhaps because of a decline in metabolic capability with ageing, older individuals are at a greater risk of metabolic deterioration and tend to develop metabolic abnormalities. Accordingly, for older individuals, it is imperative that targeted interventions, such as behavioural changes and clinical therapies, be taken to maintain metabolic health or improve metabolic deterioration.

Third, our study found that elevated ALT and UA levels are important predictors of the transition to an unfavourable weight or metabolic status. Many studies have indicated that liver enzymes are highly sensitive in predicting metabolic disorders (20, 32). A study indicated that serum ALT activity increased with increasing BMI after accounting for age and sex (33). Moreover, many studies have demonstrated that elevated UA levels are linked with obesity, insulin resistance and MetS (34). A study showed that UA levels were positively correlated with BMI after controlling for confounding factors (35).

However, the effect of elevated AST levels on specific transitions was not consistent with the above results. Elevated AST levels increased the relative risk of transitioning to an unfavourable metabolic or weight status, while elevated AST levels also increased the likelihood of recovery to a favourable metabolic or weight status. It is difficult to interpret these results at present; therefore, we need further studies to verify these results when confounding is considered fully and sample sizes are large enough.

Several strengths of this study deserve comment. This was a longitudinal study that monitored the measurements of obesity and metabolic profiles over time in an apparently healthy cohort and applied a dynamic statistical model, a multistate Markov model, so our study allowed us to completely and accurately quantify the features of possible transitions between different metabolism-weight phenotypes. To the best of our knowledge, this is the first study to explore the natural transitions of these phenotypes as a stochastic process in a general population, unlike previous studies that focused on MHO or only examined specific transitions over time. Moreover, our findings are beneficial for identifying the relatively stable or unstable phenotypes and the most likely transitions according to a complete picture of possible transitions based on a multistate Markov model; therefore, our findings may guide the planning and implementation of interventions for early prevention and even treatment to maintain health.

There were still some limitations in this study. First, the participants were only from a certain health check-up centre, and most of them were urban residents. Those younger than age 45 at baseline made up a large proportion (74.7%) of the study population. These points were likely to limit the generalization of our results to the general population. Second, only 31.12% of the participants underwent four or more follow-up visits in the study. The goodness-of-fit of the model may have decreased due to the short follow-up period. Finally, the present study did not collect sufficient information on medication use, dietary habits and other lifestyle factors due to the limitation of health check-up data, so the effects of these factors on the transitions were not examined.

## Conclusion

In conclusion, individuals with a higher initial BMI (overweight and especially obese) were more likely to progress to metabolic abnormalities and less likely to revert to metabolic health than those with a lower initial BMI (normal weight). This emphasizes that maintaining a normal and stable BMI is important for metabolic health. Additionally, the male and middle-aged groups had a higher risk of progressing to metabolic abnormalities and a lower possibility of reverting to metabolic health than the female and younger groups. This suggests that more attention should be given to males and elderly people to prevent their progression to an unhealthy metabolic status. More importantly, MHO is the most unstable phenotype and is prone to convert to MUO, a relatively stable and unfavourable state. Therefore, individuals in MHO should be given more attention, and measures should be taken in a timely manner to help them improve their weight status and prevent the deterioration of their metabolic status. Finally, individuals with elevated ALT, AST and UA levels are at an increased risk of transitioning to an unhealthy weight or metabolic status, so we should be alert to the abnormal indicators and timely take measures to maintain their normal levels to maintain a healthy weight and metabolic status.

## Data availability statement

The raw data supporting the conclusions of this article will be made available by the authors, without undue reservation.

## Ethics statement

The studies involving human participants were reviewed and approved by Ethics Committee of Dalian Medical University (Ethics Approval No. 2020 006). Written informed consent for participation was not required for this study in accordance with the national legislation and the institutional requirements.

## Author contributions

HZ screened, extracted, analyzed the data, and wrote the draft. GS designed the study and revised the manuscript. XT, DH, and GL reviewed the results, interpreted the data, and reviewed the manuscript. All authors read and approved the final manuscript.
