# Supplementary material for: Transition patterns of metabolism-weight phenotypes over time: A longitudinal study using the multistate Markov model in China
Source: Front Public Health. 2022 Dec 15;10:1026751. doi: 10.3389/fpubh.2022.1026751 (PMC9799718; doi:10.3389/fpubh.2022.1026751)
Supplement: Supplementary file 1 [file Table_1.DOCX]

**Supplementary Table 1** The transition intensities from one phenotype to another and 95% confidence interval estimated by the multistate model [q(95%CI)]

| Original phenotype | Follow-up phenotype | | | | | |
| --- | --- | --- | --- | --- | --- | --- |
|  | **MHNW** | **MHOW** | **MHO** | **MUNW** | **MUOW** | **MUO** |
| **MHNW** | -0.26  (-0.27,-0.25) | 0.11  ( 0.10, 0.12) | 0 | 0.15  ( 0.14, 0.16) | 0 | 0 |
| **MHOW** | 0.20  ( 0.18, 0.21) | -0.64  (-0.67,-0.61) | 0.07  ( 0.06, 0.08) | 0 | 0.38  ( 0.35, 0.40) | 0 |
| **MHO** | 0 | 0.30  ( 0.26, 0.35) | -0.86  (-0.95,-0.78) | 0 | 0 | 0.56  ( 0.49, 0.63) |
| **MUNW** | 0.63  ( 0.59, 0.68) | 0 | 0 | -0.82  (-0.86,-0.78) | 0.18  ( 0.16, 0.20) | 0 |
| **MUOW** | 0 | 0.44  ( 0.41, 0.48) | 0 | 0.10  ( 0.09, 0.11) | -0.63  (-0.66,-0.60) | 0.09  ( 0.08, 0.10) |
| **MUO** | 0 | 0 | 0.27  ( 0.24, 0.31) | 0 | 0.16  ( 0.14, 0.18) | -0.43  (-0.47,-0.39) |

MHNW, metabolically healthy normal weight; MHOW, metabolically healthy overweight; MHO, metabolically healthy obesity; MUNW, metabolically unhealthy normal weight;MUOW, metabolically unhealthy overweight；MUO, metabolically unhealthy obesity.

**Supplementary Table 2** The effects of some factors on transitions from one phenotype to another [hazard ratios (95% CIs)].

| Factors | *MHNW→MHOW* | *MHNW→*  *MUNW* | *MHOW→*  *MHO* | *MHOW→*  *MUOW* | *MHO→*  *MUO* | *MUNW→*  *MUOW* | *MUOW→*  *MUO* |
| --- | --- | --- | --- | --- | --- | --- | --- |
| Sex^a^ | 0.63 ^*^  (0.55,0.73) | 0.68 ^*^  (0.58,0.79) | 1.24  (0.91,1.70) | 0.77 ^*^  (0.66,0.90) | 0.96  (0.69,1.32) | 0.83  (0.64,1.08) | 1.07  (0.80,1.45) |
| Age^b^ | 0.82 ^*^  (0.68,0.99) | 1.79^*^  (1.53,2.09) | 0.71  (0.49,1.02） | 1.24^*^  (1.07,1.44) | 1.14  (0.84,1.55) | 0.85  (0.66,1.10) | 0.76 ^*^  (0.58,1.00) |
| ALT^c^ | 1.57 ^*^  (1.22,2.01) | 1.16  (0.88,1.53) | 1.07  (0.72,1.58) | 1.21 ^*^  (1.00,1.47) | 1.28  (0.96,1.70) | 1.55 ^*^  (1.13,2.14) | 0.90  (0.66,1.22) |
| AST^d^ | 1.23  (0.76,1.99) | 1.81 ^*^  (1.03,3.19) | 0.55  (0.16,1.78) | 0.88  (0.57,1.36) | 1.11  (0.61,2.02) | 0.50  (0.19,1.30) | 1.724^*^  (1.03, 2.88) |
| SCr^e^ | 0.78  (0.52,1.16) | 1.25  (0.91,1.72) | 1.50  (0.75,3.00) | 1.23 (0.79,1.91) | 1.15  (0.52,2.56) | 0.94  (0.48,1.84) | 1.14  (0.47, 2.75） |
| UA^f^ | 1.29 ^*^  (1.01,1.64) | 1.374 ^*^  (1.08,1.76) | 1.37  (0.94,1.96) | 1.06  (0.89,1.27) | 1.76 ^*^  (1.32,2.34) | 1.25  (0.90,1.73) | 1.20  (0.91,1.60) |
| Factors | *MHOW→*  *MHNW* | *MUNW→*  *MHNW* | *MHO→*  *MHOW* | *MUOW→*  *MHOW* | *MUO→*  *MHO* | *MUOW→*  *MUNW* | *MUO→*  *MUOW* |
| Sex | 2.27^*^  (1.90,2.71) | 1.19 ^*^  (1.02,1.40) | 1.84 ^*^  (1.32,2.56) | 1.17 ^*^  (1.00,1.37) | 1.17  (0.84,1.62) | 1.840^*^  (1.40,2.43) | 1.23  (0.87,1.74) |
| Age | 0.71^*^  (0.58,0.86) | 0.71 ^*^  (0.60,0.83) | 1.13  (0.77,1.64) | 0.81 ^*^  (0.69,0.94) | 0.65 ^*^  (0.47,0.89) | 0.90  (0.69,1.18) | 1.14  (0.85,1.53) |
| ALT | 1.11  (0.87,1.42) | 0.72 ^*^  (0.55,0.93) | 0.66  (0.42,1.02) | 0.91  (0.76,1.08) | 0.72 ^*^  (0.54,0.97) | 0.58^*^  (0.39,0.85) | 0.69^*^  (0.50,0.94) |
| AST | 1.06  (0.57,1.97) | 1.87 ^*^  (1.14,3.08) | 1.78  (0.76,4.16) | 0.93  (0.62,1.41) | 0.63  (0.34,1.16) | 2.48 ^*^  (1.31,4.68) | 1.36  (0.83,2.24) |
| SCr | 0.91  (0.62,1.34) | 0.82  (0.55,1.21) | 1.09  (0.52, 2.29) | 1.17  (0.76,1.81) | 0.58  (0.21,1.57) | 0.90  (0.39, 2.07) | 1.81  (0.98, 3.33) |
| UA | 0.96  (0.75,1.22) | 0.91  (0.72,1.15) | 1.12  (0.77,1.64) | 0.80 ^*^  (0.67,0.96) | 1.11  (0.83,1.50) | 0.84  (0.60,1.17) | 0.83  (0.61,1.14) |

**Abbreviations:** ALT, alanine aminotransferase; AST, aspartate aminotransferase; SCr, serum creatinine; UA, uric acid. MHNW, metabolically healthy normal weight; MHOW, metabolically healthy overweight; MHO, metabolically healthy obesity; MUNW, metabolically unhealthy normal weight;MUOW, metabolically unhealthy overweight；MUO, metabolically unhealthy obesity.

**Note:** **P*＜0.05; a. Sex, women v.s. men；b. Age，middle-aged group(＞45 years old) vs. young group (≤45years old)； c.ALT, the elevated v.s. the normal; d. AST, the elevated v.s. the normal; e. SCr, the elevated v.s. the normal; f.UA, the elevated v.s. the normal.

**Supplementary Table 3** The estimated transition intensities for each subgroup

| Transition | Young male group^a^ | | Middle-aged male group^b^ | | Young female group^c^ | | Middle-aged female group^d^ | |
| --- | --- | --- | --- | --- | --- | --- | --- | --- |
|  | Estimates | 95% CI | Estimates | 95% CI | Estimates | 95% CI | Estimates | 95% CI |
| MHNW→MHOW | 0.16 | ( 0.14, 0.18) | 0.14 | (0.11,0.18) | 0.10 | (0.09,0.11) | 0.08 | (0.06,0.10) |
| MHOW→MHNW | 0.13 | ( 0.11, 0.15) | 0.11 | (0.09,0.15) | 0.32 | (0.29,0.35) | 0.20 | (0.16,0.25) |
| MHOW→MHO | 0.07 | ( 0.06, 0.09) | 0.06 | (0.04,0.08) | 0.09 | (0.07,0.11) | 0.04 | (0.02,0.08) |
| MHO→MHOW | 0.23 | ( 0.18, 0.28) | 0.28 | (0.19,0.43) | 0.47 | (0.37,0.60) | 0.36 | (0.21,0.61) |
| MUNW→MUOW | 0.24 | ( 0.20, 0.30) | 0.18 | (0.14,0.24) | 0.16 | (0.13,0.20) | 0.15 | (0.12,0.21) |
| MUOW→MUNW | 0.08 | ( 0.06, 0.10) | 0.08 | (0.06,0.11) | 0.18 | (0.14,0.23) | 0.13 | (0.09,0.18) |
| MUOW→MUO | 0.09 | (0.08,0.11) | 0.07 | (0.06,0.09) | 0.10 | (0.07,0.13) | 0.07 | (0.05,0.11) |
| MUO→MUOW | 0.13 | (0.10,0.16) | 0.18 | (0.14,0.23) | 0.24 | (0.18,0.33) | 0.18 | (0.12,0.29) |
| MHNW→MUNW | 0.20 | (0.17,0.23) | 0.33 | (0.27,0.40) | 0.12 | (0.11,0.13) | 0.23 | (0.19,0.27) |
| MUNW→MHNW | 0.63 | (0.54,0.73) | 0.49 | (0.41,0.60) | 0.81 | (0.73,0.90) | 0.54 | (0.46,0.65) |
| MHOW→MUOW | 0.41 | (0.37,0.45) | 0.48 | (0.420,0.56) | 0.30 | (0.27,0.35) | 0.41 | (0.33,0.50) |
| MUOW→MHOW | 0.45 | (0.41,0.50) | 0.36 | (0.31,0.42) | 0.56 | (0.48,0.65) | 0.47 | (0.38,0.58) |
| MHO→MUO | 0.57 | (0.49,0.68) | 0.73 | (0.54,0.99) | 0.53 | (0.40,0.69) | 0.38 | (0.22,0.65) |
| MUO→MHO | 0.28 | (0.23,0.33) | 0.23 | (0.17,0.31) | 0.38 | (0.28,0.51) | 0.17 | (0.10,0.31) |

MHNW, metabolically healthy normal weight; MHOW, metabolically healthy overweight; MHO, metabolically healthy obesity; MUNW, metabolically unhealthy normal weight;MUOW, metabolically unhealthy overweight；MUO, metabolically unhealthy obesity. a: young male group (≤45years old); b:middle-aged male group(＞45 years old) ; c: young female group (≤45years old); d: middle-aged female group(＞45 years old).

**Supplementary Table 4** The mean sojourn time for different phenotypes in each subgroup

| States | Young group | | | Middle-aged group | | |
| --- | --- | --- | --- | --- | --- | --- |
|  | Estimate (years) | Standard errors | 95% CI | Estimate (years) | Standard errors | 95% CI |
| **Male** |  |  |  |  |  |  |
| MHNW | 2.76 | 0.13 | (2.52,3.02) | 2.14 | 0.16 | (1.85,2.48) |
| MHOW | 1.64 | 0.07 | (1.52,1.77) | 1.54 | 0.09 | (1.37,1.73) |
| MHO | 1.25 | 0.08 | (1.09,1.43) | 0.98 | 0.12 | (0.77,1.26) |
| MUNW | 1.15 | 0.07 | (1.02,1.29) | 1.48 | 0.12 | (1.27,1.74) |
| MUOW | 1.61 | 0.07 | (1.48,1.75) | 1.95 | 0.11 | (1.74,2.18) |
| MUO | 2.48 | 0.16 | (2.19,2.81) | 2.45 | 0.25 | (2.01,3.00) |
| **Female** |  |  |  |  |  |  |
| MHNW | 4.57 | 0.15 | (4.29,4.88) | 3.24 | 0.23 | (2.83,3.71) |
| MHOW | 1.41 | 0.06 | (1.30,1.52) | 1.55 | 0.12 | (1.33,1.81) |
| MHO | 1.01 | 0.09 | (0.84,1.21) | 1.35 | 0.25 | (0.94,1.96) |
| MUNW | 1.03 | 0.05 | (0.94,1.30) | 1.43 | 0.11 | (1.23,1.67) |
| MUOW | 1.19 | 0.07 | (1.07,1.33) | 1.51 | 0.13 | (1.28,1.78) |
| MUO | 1.62 | 0.16 | (1.32,1.97) | 2.81 | 0.49 | (1.99,3.96) |

MHNW, metabolically healthy normal weight; MHOW, metabolically healthy overweight; MHO, metabolically healthy obesity; MUNW, metabolically unhealthy normal weight;MUOW, metabolically unhealthy overweight；MUO, metabolically unhealthy obesity.
